# Editorial: Community series - innovative approaches in diagnosis of emerging/re-emerging infectious diseases, volume II

**DOI:** 10.3389/fmicb.2023.1193841

**Published:** 2023-05-04

**Authors:** Toshana Foster, Svetlana Khaiboullina

**Affiliations:** ^1^Faculty of Medicine and Health Sciences, School of Veterinary Medicine and Science, Wolfson Centre for Global Virus Research, The University of Nottingham, Loughborough, United Kingdom; ^2^Department of Microbiology, University of Nevada, Reno, Reno, NV, United States

**Keywords:** emerging infection, diagnostics, innovative approaches, rapid diagnostics, innovative methods, protozoan, virus, fungus

Emerging and re-emerging infections remain a rising burden on public health and global economies. Several outbreaks of emerging infectious diseases and notably, the COVID-19 pandemic have had significant impacts throughout the 21^st^ century. Across multiple countries, each infection outbreak made a remarkable influence on population health and healthcare facilities, despite recent improved medical and diagnostic advances in countering disease epidemics: a high mortality rate was characteristic of the Ebola virus disease epidemic in 2012–2016 (Li et al., 2016; Forna et al., 2020), the teratogenic effects of Zika virus (ZIKV) were reported during the outbreak in French Polynesia in 2013–2014 and Brazil in 2015–2016 (Gilbert et al., 2023), and the highly contagious nature of the severe acute respiratory syndrome coronavirus 2 (SARS-CoV-2) contributed to its rapid spread and the global pandemic in 2020 (Mohapatra et al., 2020).

The risk of emerging and re-emerging pathogens with epidemic and pandemic potential is expected to remain high (Baker et al., 2022). The rapid spread of these pathogens is driven by the variable dynamic balance/imbalance between the pathogen, host and environment, influenced by demographic change as a catalyst for increased transmission dynamics, changes in reservoir species range and density due to climate change particularly for vector-borne disease spread (Brashares et al., 2004; Viboud et al., 2006; McDermott, 2022), drug resistance and declining immunity due to the aging global population (Schaible and Kaufmann, 2007; Dropulic and Lederman, 2016; Harpaz et al., 2016; Corey et al., 2022). Understanding the drivers and contributors of disease spread and pathogenesis is important when designing successful measures of control. Given that emerging and re-emerging and diseases are characterized by acute onset and rapid development of symptoms, early and reliable diagnosis of infection is essential to reduce mortality and to select the most effective treatment strategy. The fast development of ultra-high throughput systems for detecting SARS-CoV-2 (Biosearchtech, 2022) and the development of the point-of-care test that allows healthcare providers to reduce the time for diagnosis (Song et al., 2021) are a reflection of the recent, rapid diagnostics advances made that can influence outbreak dynamics.

The aim of this Research Topic was to facilitate the sharing of innovative approaches in diagnosing emerging and re-emerging diseases. We focused on highlighting the importance and urgency of developing novel, rapid and more accurate diagnostic tools. The scope of the four original articles published under this topic included research on identifying diagnostic targets for fungal, bacterial, and viral pathogens.

The original article by Hsiao et al. elucidate the universal optimal cut-off values used in *Aspergillus*-specific antibody tests as diagnostic tools for the three classified types of aspergillosis, allergic bronchopulmonary aspergillosis (ABPA), chronic pulmonary aspergillosis (CPA), and invasive aspergillosis (IA). Aspergillus infection is frequently diagnosed in immunocompromised individuals (Gletsou et al., 2018). In immunocompromised patients, the disease could be in an invasive form with fatality rates as high as 50–85% and that can remain high even after specific treatment (Lin et al., 2001; Lowes et al., 2017). Therefore, early diagnosis of the disease could improve patient survival, where currently a single test does not exist to definitively confirm *Aspergillus* infection. The diagnosis of CPA is based on detecting fungus-specific IgG (Shin et al., 2014). However, substantial differences in anti-Aspergillus antibodies based on ethnicity, geographic location, and frequency of exposure have been reported (Rayens et al., 2022). This makes it challenging to establish a biological reference range for aspergillosis and thus the universal cut-off values for Aspergillus antibody tests. In the current study, Hsiao et al. aimed to develop the cut-off values for Aspergillus-specific antibodies in the Taiwanese population and compare and report on geographic variations. The authors collected serum samples from 118 controls and 128 patients with IA, CPA, and ABPA between June 2018 to September 2021. All serum samples were probed for *Aspergillus (A.) fumigatus* and *A. niger*-specific IgG and IgE using ImmunoCAP technology. Optimal cut-offs were determined using receiver operating characteristic curve (ROC) analysis.

The authors found that CPA patients had the highest *A. fumigatus*-specific IgG levels compared to other forms of aspergillosis. In contrast, patients with ABPA had the highest *A. fumigatus*-specific IgE and *A. niger*-specific IgG and IgE serum levels compared to IA, with *A. niger*-specific IgE demonstrated in this study to be a potential a diagnostic tool for ABPA. Based on collected data, the authors determined the optimal cut-off of *A. fumigatus* and *A. niger*-specific IgG for patients with CPA and ABPA in the Taiwanese population. The authors also described that the cut-off differences for IgG antibodies to *A. fumigatus* were higher in patients living in different eco-climatic zones. These findings infer that geographic variations may affect antibody levels in patients and suggest that further work is needed to establish reference ranges across affected countries.

The genotyping of *Chlamydia (Ch.) trachomatis* (CT) and the spatiotemporal spread of lymphogranuloma venereum (LGV) in Madrid were the focus of the original manuscript by Martinez-Garcia et al. LGV, which clinically commonly presents with inguinal femoral lymphadenopathy, proctitis, proctocolitis and ulcers is increasingly endemic in vulnerable populations in Europe (Stoner and Cohen, 2015; Control, 2019). However, our understanding of the disease epidemic remains incomplete due to the limited molecular epidemiology data on *Ch. trachomatis*, the causative agent of a broad spectrum of diseases (Dean et al., 1991; Rodríguez-Domínguez et al., 2014). LGV is caused by the L-genotypes (L1, L2, and L3) of CT (Ceovic and Gulin, 2015) and current the molecular epidemiology of the LGV epeidemic is based on sequence analysis of the *ompA* and *ompA-pmpH* genes. However, this test has limited discriminatory power across the different genotypes of LGV. The authors aimed to use multilocus sequence typing (MLST) methods and a novel combination of molecular markers with high diversity derived from variable genes of L-genotypes genomes to improve the specificity of *Ch. trachomatis* genotyping in Madrid. Martinez-Garcia et al. selected four genes, *CTLon_0054, CTLon_0087, CTLon_0243, and CTLon_0301*, in addition to *ompA*, to complete the genotype analysis of *Ch. trachomatis*. *In silico* and experimental studies were performed to compare the previously described MLST schemes and genes selected by authors. The diversity analysis approach tested by authors identified higher diversity of *Ch. Trachomatis* than previously reported and identified 3 main transmission chains. Subsequent spacio-temporal analysis revealed the major cluster was characterized by high diversification of *Ch. Trachomatis* genome and an active transmission chain. The authors also detected the L2b genome identical to the epidemic's origin, suggesting re-introductions or low screening rates in the studied population. Overall, this study proposes new methods to monitor sexual networks with higher precision, offers the possibility of differentiating between reinfection and persistence and identifying hotspots that could be crucial for contact tracing.

In a study by Liu et al., the clinical validity of the combination of gut microbiome data and common clinical indicators was analyzed as a predictor of myocardial infarction in young males. ST-segment elevation myocardial infarction (STEMI) in young male patients could indicate high heart attack risk (Zhang et al., 2016). Studies have demonstrated that changes in the gut microbiome could contribute to the incidence of coronary artery disease (CAD) (Koeth et al., 2013; Yoshida et al., 2018; Liu et al., 2019). This hypothesis was further applied to the pathogenesis of STEMI, a severe form of CAD in young males. The authors, Liu et al., therefore aimed to develop an early risk prediction model based on a comprehensive analysis of the gut microbiome in young male patients with STEMI combined with clinical data to address this hypothesis. They conducted the Illumina sequencing of 16S rRNA of the gut microbiome of 41 age and gender-matched STEMI patients and 40 non-CAD controls. The collected data was used to evaluate the efficacy and practicability by generating a nomogram and corresponding web page with K-fold cross-validation, calibration curves and decision curve analysis (DCA). The authors demonstrated a substantially decreased α and β diversity and significantly altered gut microbiome in the young male patients with STEMI compared to controls. Also, the STMEI patients had higher body mass index (BMI), systolic blood pressure (SBP), triglyceride (TG), alanine aminotransferase (ALT), and aspartate aminotransferase (AST) parameters, while blood urea nitrogen (BUN) was lower compared to the control. Additionally, authors found that BMI and SBP had a positive correlation with *Streptococcus* and *[Ruminococcus]* and a negative correlation with *Prevotella* and *Megasphaera*, implying that alterations in microbiome composition and abundances could play a role in the biochemical and metabolic changes in young male STEMI patients. Analysis of the calibration curves of the microbiome prediction model demonstrated consistency between the actual and predicted probabilities, with an indication that the microbiome model was superior to the clinical model. The authors concluded that combining data on the gut microbiome and clinical signs could be a valid and reliable non-invasive tool to predict STEMI in young males.

The original manuscript by Wang et al. addressed the urgent need for a sensitive and cost-effective novel approach to identify subtypes of influenza A viruses (IAVs). There are several subtypes of IAVs based on the expression of surface proteins hemagglutinin (HA) and neuraminidase (NA) (Schulze et al., 2010). The IAV variants emerge as a result of reassortment between genes coding for various HA and NA genes (Bhoumik and Hughes, 2010). The emergence of these new IAVs subtypes could be linked to different clinical manifestations of the disease. H1N1 (pH1N1) genotype usually causes a mild flu infection (Huo et al., 2019). In contrast, H5N1 viruses are highly pathogenic (HP), with a mortality rate of 60% (Yin et al., 2013). A rapid, sensitive, and cost-effective test which can differentiate across H5N1 and H1N1 subtypes, which present with similar symptoms upon initial infection, could aid in the management of influenza pandemic and control of HP infection spread. To develop a test that improved upon current virus culture, serology and genetic testing methods, the authors developed an approach using the spectral tool, Fluorescence resonance energy transfer (FRET), that is widely used for intermolecular interaction studies. They designed a homogeneous fluorescence method for dual detection of HA proteins of H1N1 and H5N1 influenza viruses—an aptasensor for HA using FRET strategy combined with DNase I-assisted cyclic enzymatic signal amplification. Fluorescent-labeled HA aptamers of H1N1 and H5N1 IAVs were designed and used as probes. Graphene oxide (GO) was used as a FRET acceptor and protected aptamers from DNase I cleavage. Increased sensitivity of the test was based on the amplification of fluorescence signal by liberated aptamers and HA proteins after DNase I digest. This test system was approximately 20-fold greater in sensitivity than traditional fluorometric methods without amplification. The authors also state no cross-reaction between H1N1 and H5N1 IAV subtypes with good reproducibility and stability of the aptasensor probes. This tool could be applied to the clinical setting to aid in the typing and early detection of influenza virus and surveillance for early pandemic preparedness efforts.

This Research Topic highlights the novel approaches in the detection of emerging infections. The development of novel diagnosis methods addresses the most urgent needs, which are sensitivity, specificity, speed of detection and affordability. The knowledge attained from the articles on this Research Topic could facilitate the development of diagnostic tools for the detection of emerging infections. This research is essential and urgently needed to improve our preparedness for future pandemics.

We are grateful to the authors and reviewers for their valuable contribution to the research and discussion that will leverage new frameworks for infectious disease diagnostics and control that responds to the increasing risk from emerging and re- emerging pathogens.

## Author contributions

TF: conceptualization, investigation, and writing—original draft. SK: writing–original draft and writing–review and editing. All authors contributed to the article and approved the submitted version.
